# Corundum Wheels

**Published:** 1851-04

**Authors:** 


*Corundum Wheels.*—Mr. Francis Arnold, dental and surgical instrument manufacturer, of Baltimore, has recently imported from England an assortment of corundum wheels, varying in diameter from half an inch to four inches. He has also slabs, varying in shape and thickness, especially designed for mechanical dentistry. To those who have never used corundum wheels, it may be necessary to state that they cut porcelain about four times as fast as an emery wheel, and leave a much smoother surface. As they have been used in our laboratory for about two years, we can certify to their superiority over any kind of grinding wheel which has come under our notice.

